# Supplementary material for: The Penn Medicine BioBank: Towards a Genomics-Enabled Learning Healthcare System to Accelerate Precision Medicine in a Diverse Population
Source: J Pers Med. 2022 Nov 29;12(12):1974. doi: 10.3390/jpm12121974 (PMC9785650; doi:10.3390/jpm12121974)
Supplement: Supplementary file 1 [file jpm-12-01974-s001.zip › PMBB_Banner_Author_List_and_Contribution_Statements_07252022.pdf]

Penn Medicine BioBank Banner Author List and Contribution Statements

<u>PMBB Leadership Team</u>
Daniel J. Rader, M.D., Marylyn D. Ritchie, Ph.D., Michael D. Feldman M.D.

Contribution: All authors contributed to securing funding, study design and oversight. All authors reviewed the final version of the manuscript.

<u>Patient Recruitment and Regulatory Oversight</u>
JoEllen Weaver, Afiya Poindexter, Ashlei Brock, Khadijah Hu-Sain, Yi-An Ko

Contributions: JW manage patient recruitment and regulatory oversight of study. AP, AB, KH, YK recruitment and enrollment of study participants.

<u>Lab Operations</u>
JoEllen Weaver, Meghan Livingstone, Fred Vadivieso, Ashley Kloter, Stephanie DerOhannessian, Teo Tran, Linda Morrel, Ned Haubein, Joseph Dunn

Contribution: JW, ML, FV, SD oversight of lab operations. ML, FV, AK, SD, TT, LM perform sample processing. NH, JD are responsible for sample tracking and the laboratory information management system.

<u>Clinical Informatics</u>
Anurag Verma, Ph.D., Colleen Morse, M.S., Marjorie Risman, M.S., Renae Judy, B.S.

Contribution: All authors contributed to the development and validation of clinical phenotypes used to identify study subjects and (when applicable) controls.

<u>Genome Informatics</u>
Anurag Verma Ph.D., Shefali S. Verma, Ph.D., Yuki Bradford, M.S., Scott Dudek, M.S., Theodore Drivas, M.D., PH.D.

Contribution: A.V., S.S.V. are responsible for the analysis design and infrastructure needed quality control genotype and exome data. Y.B. performed the analysis. T.D. and A.V. provide variant and gene annotations and their functional interpretation of variants.
